# “*Walking around the golf course is the exercise you need”*: Exploring the acceptability of a golf on referral scheme amongst participants through post-programme focus groups

**DOI:** 10.1186/s12875-026-03180-1

**Published:** 2026-01-27

**Authors:** Samuel J Warne, Lynsey R Brown, Kevin Barker, Sharon A Carstairs, Kathryn Burns Cunningham, Sandhya Duggal, Iain Evans, Allan Martin, Andrew Murray, Gozde Ozakinci, Andrew J Williams, Frank Sullivan

**Affiliations:** 1 Population & Behavioural Science Division, School of Medicine, University of St Andrews, St Andrews, KY16 9TF UK; 2 The R&A, St Andrews, UK; 3 School of Health Sciences, University of Dundee, Dundee, UK; 4 Education Division, School of Medicine, University of St Andrews, St Andrews, UK; 5 Fife Golf Trust, Kirkcaldy, UK; 6 Professional Golfers Association, Gleneagles, UK; 7 European Tour Health & Performance Institute, European Tour Group, Surrey, UK; 8 Division of Psychology, Faculty of Natural Sciences, University of Stirling, Stirling, UK; 9 Scottish Collaboration for Public Health Research and Policy, University of Edinburgh, Edinburgh, UK

**Keywords:** Social prescribing, Physical activity, Exercise referral, Community connection, Focus groups, Thematic analysis

## Abstract

**Background:**

Physical inactivity has significant adverse consequences for health and wellbeing, yet its ubiquity in higher income countries continues to be a major issue. Participation in sport is one possible solution to reducing inactivity, with evidence showing its ability to increase physical activity levels, improving physical and mental health, as well as social connections. Recent research has suggested that the benefits of sports, such as golf, could be achieved through social prescribing schemes, connecting inactive individuals to local activity opportunities. However, it is unclear how this connection pathway between community health settings and golf clubs may work in practice. A pilot Golf for Health scheme was implemented in Fife, Scotland. The present research aimed to assess the acceptability of this programme and its connection pathways, exploring this amongst participants’ experiences.

**Methods:**

Healthcare practices and allied professionals in Fife were invited to take part in the pilot study, connecting inactive individuals with five local golf clubs offering a free-of-charge ‘Golf for Health’ programme. Following the pilot, two semi-structured focus groups (*n* = 9) were conducted with participants who completed the programme. These aimed to explore how well the intervention was received and the extent to which it met participant needs, as well as the initial connection experience. Transcripts were analysed using thematic analysis.

**Results:**

Three overarching themes were developed, representing the barriers and facilitators to engagement; the connection pathway and sign-up process; and the participants’ programme experience. Participants enjoyed the programme, appreciated the chance to learn golf and make new friends in a welcoming environment; however, there were concerns regarding the connection pathways and sign-up process.

**Conclusions:**

Overall responses were positive, with reports that participation was generally beneficial. ‘Golf for Health’ may offer an accessible and social introduction to golf, subsequently providing opportunity for long-term health and wellbeing benefits. More work is needed on developing connection pathways that are acceptable and feasible in practice.

**Supplementary Information:**

The online version contains supplementary material available at 10.1186/s12875-026-03180-1.

## Introduction

Regular physical activity can enhance an individual’s physical, mental, cognitive and social wellbeing [1]. However, despite the benefits, the prevalence of inactivity both in the UK and globally is high, with nearly 40% of the adult population of high-income Western countries identified as physically inactive [2]. In this paper, physical activity refers to the World Health Organization (WHO) recommendations, which state that to be deemed physically active and to gain the aforementioned health benefits, an adult should achieve at least 150 min of moderate intensity (or 75 min of vigorous intensity) aerobic activity each week, noting that additional activity confers further health benefits [1, 3]. Recognising the role that regular physical activity and exercise can have on an individual’s health and wellbeing, there has been a steady move toward the encouragement of this for a decade [4]. Nevertheless, it is widely understood that achieving the recommended 150 min of moderate-intensity physical activity a week is difficult for many people, especially those living with long-term health conditions [2, 5, 6].

Acknowledging this, the 2019 UK Chief Medical Officers’ Physical Activity Guidelines began by stating that “If physical activity were a drug, we would refer to it as a miracle cure, due to the great many illnesses it can prevent and help treat” [7]. Targeting a nation with *‘More People*,* More Active*,* More Often’*, policies have been implemented in Scotland specific to this notion - represented by the National Physical Activity for Health Framework [8], Physical Activity Outcomes Framework [9], and Physical Activity Referral Standards [10]. Supported by the recently published Population Health Framework [11], the National Physical Activity for Health Framework calls for an Active Health and Social Care system. This involves integrating the NHS Physical Activity Pathway into routine healthcare, and developing partnerships with local physical activity providers.

The inclusion of physical activity in health interventions can be pivotal for promoting and maintaining good health [12]. Connecting individuals with community-based physical activity has potential to be beneficial to all parties [13], as not only will the individual have the chance to improve their own health whilst doing something that they enjoy, but it has also been shown that those who are connected to these opportunities require fewer GP appointments following their engagement [14]. Therefore, it is essential that connection and referral pathways and the opportunities themselves are as efficient and effective as possible, so to best aid the individual, raise physical activity rates, and in doing so potentially ease some of the strain on health services.

Social prescribing from community health settings to a range of activities is one potential route to address this problem [15]. These are typically non-medical, but prescribed by community health and social care professionals, enabling patients to attain achievable health goals [16, 17]. To facilitate the connection phase of social prescribing, over 3,500 Community Link Workers (CLW) are now employed in England alone, connecting over 2.5 million people to a range of local services [18]. Meanwhile, Scotland have 300 employed CLWs, accessible to 80% of the nation’s GP practices [19, 20]. Scottish CLW services operate primarily within areas where there is greatest need, based on deprivation and health inequalities [20]. Broadly speaking, the role of the CLW is to act as a non-clinical member of a GP, working closely with patients and individuals in need, to connect them to a local service that is both of interest and benefit to them, helping to address their practical, social and emotional needs [16, 21, 22]. Furthermore, it should be noted that there is scepticism surrounding the use of the term ‘prescribing’ in this context, such that it has the potential to eradicate the autonomous and communal nature of the programmes that patients are connected with, instead implying a sense of authoritarian compliance [23].

A scoping review by Cunningham et al. identified five methods of connection from primary care to community-based physical activity opportunities [15]. The simplest of these, is the direct ‘Active Signposting’ route, whereby a Primary Care Professional actively recommends a local community-based physical activity opportunity, providing the patient with information. Alternatively, four ‘Indirect Routes’ were established, these representing those occasions where intermediary facilitation is required, using a combination of active signposting and direct referral between the primary care professional and the facilitator, toward the physical activity opportunity itself [15].

The governing body of Golf (The R&A) have actively advocated its potential use for health promotion [24]. Golf is a moderate-intensity, aerobic sport that presents individuals with the opportunity for a social activity that can be played across the lifespan. This therefore enables those who play it to achieve potential risk reductions associated with regular physical activity, such as a 35% reduced risk of cardiovascular disease and 30% reduced risk of all-cause mortality [25–27]. With additional physical, mental and cognitive stimulation compared to walking [28–30], golf has been linked to positive outcomes in both physical and mental health [25, 26]. It has previously been proposed that golf could be recommended to healthy older adults for the prevention of cardiovascular disease [29]. Research has also linked golf participation with significantly greater self-reported Quality of Life, most recently shown in a post-operative sample [31]. This may be due to not only the links with greater physical health [25, 32], but also the positive associations between golf and psychological wellbeing [33]. The cumulative possible benefits of golf participation may amount to why golf participation was found to be associated with an added 5 years of life expectancy [34], with previous trial data also supporting the positive health benefits of golf [35]. An additional benefit and accessible advantage is that golf has many forms, whether the traditional 18 hole format, shorter course, or just a driving range, all of which are rated ≥ 3.5 METs (metabolic equivalents of task, where one MET represents the rate of energy expenditure at rest) on the updated MET Compendium [36]. The significance of this relates back to the World Health Organization guidelines, as they suggest a target of at least 150 min of moderate intensity activity per week, where moderate activities are denoted as those with a MET rating of 3.0–6.0 – and therefore golf in any of these formats would be adequate intensity to meet the recommendations.

A collaborative team of researchers from multiple Scottish academic institutions, as well as stakeholders including the R&A, DP World (European) Tour, Scottish Golf and Fife Golf Trust, have been working to utilise the prominent accessibility of golf in Scotland. With over 550 golf courses in Scotland alone (2nd most per capita globally; [37]), it is acknowledged that this provides opportunities for increased potential for improving physical activity connections in this context. Latest figures from the R&A’s Global Golf Participation report suggest that up to 17.5% of the Scottish population played golf in 2024 [38].

A cost-free ‘Golf for Health’ programme was designed for people with limited or no golf experience, who were not meeting the recommended guidelines for physical activity participation, with a goal to remove barriers to physical activity and provide another local physical activity opportunity for connection by local healthcare professionals. A two-phase co-design approach was taken in the conceptualisation of the programme, with collaborative input from key stakeholders (i.e. Healthcare Professionals, Link Workers, Golf Club Staff, Potential Participants), resulting in greater awareness of the desired implementation methods, connection pathways and ongoing support schemes – as reported elsewhere [39]. Following a successful pilot of this golf for health programme, which included both Autumn and Spring iterations, the aim of the present study was to explore the acceptability of the ‘Golf for Health’ social prescribing scheme for participants, their experiences and the relevant connection pathways.

## Methods

This project received ethical approval from the University of St Andrews School of Medicine Ethics Committee in March 2022 (MD16041), and NHS Research Ethics Committee (REC: 22/SC/0061). Data collection took place across 2023. All participants were fully informed, signed consent forms accordingly prior to their participation in the focus groups, and were given further opportunity to ask questions before recordings began.

### Programme

As briefly described, the Golf for Health programme was a free, socially-prescribed scheme that entailed eight-weeks of group coaching sessions at five local clubs across Fife, Scotland. To be eligible for participation, individuals had to be 18 years or older; had not played golf for at least 2 years prior to the programme beginning; and were not achieving the 150 min World Health Organization guideline (this physical inactivity was self-assessed). There were also some exclusion criteria, in line with American College of Sports Medicine (ACSM) guidance [40], including: unstable angina; severe memory impairment or dementia; those in palliative care; severe osteoporosis; and severe psychological disorders (i.e. mania or schizophrenia). Initially, connection to the programme could be made via GP practice or Healthcare Professional – however these possibilities required expansion. Overall, 66 participants took part in this Golf for Health programme (Spring, *n* = 34; Autumn, *n* = 32). In terms of demographics, the cohort comprised of 34 males and 32 females, with ages ranging from 18 to 79 years old (M = 54).

To keep the sessions as accessible as possible, not only was the tuition (delivered by the resident professional) free of charge, but all equipment was provided for them, and there were no dress-codes. Participants were also to be invited into the clubhouse after each session, to try and foster a sense of belonging into the golf-club community setting. The approaches taken to accessibility of the sessions were multifaceted, as it is noted that just making a physical activity opportunity free may increase initial interest, but does not guarantee sustained adherence – especially in the context of golf, and where health conditions are concerned [41–43].

The coaches at each location were told that sessions were targeted for beginner golfers, and should be framed to teach participants the foundations of the game. Every session also had to include aerobic activity, with 30-minutes of walking being incorporated within each. Overall, average session attendance was 69.9% (Spring: 71.6%, Autumn: 68.3%), aligning almost exactly with the average attendance levels reported in a systematic review of adherence to community based group exercise interventions for older people [44].

### Participants

Participants were purposively sampled, such that they comprised only of those who had participated in the Golf for Health programme. This was the only inclusion criteria for the present study, therefore aligning with the criteria for programme participation: An invitatation to participate in the evaluation was given to all participants of the programme, who could choose from either a focus group, or an evaluation survey (reported elsewhere).

A total of nine participants took part across the two focus groups, with ages ranging from 42 to 77 years (M = 64.4). Seven participants were female and two were male. Representation was achieved from three of the five participating golf club locations. All participants reported living with at least one long-term health condition. The sample also reflected a diversity of connection pathways into the programme, providing a breadth of perspectives on engagement and access. Those who participated in the focus groups were rewarded with a £15 voucher as recompense for their time.

### Materials, design & procedures

Two focus groups were conducted in total, one after the Autumn/Winter iteration, and a second after the Spring iteration of the programme. Each focus group lasted approximately 30–40 min and was held in person at one of the golf club locations, a relaxed environment which would be more familiar than a University space to the participants. The same researcher (LB) led each focus group session of up to five participants, with group sizes kept small so that everybody had a chance to relay the information they wished to without feeling rushed, outspoken or under any pressure [45]. One of the focus group sessions also had a coach participate.

The focus group discussions were semi-structured in nature, with a topic guide (Supplementary File 1) utilised to ensure that the primary points of interest were covered, in addition to anything that the participants wished to add. The advantage of a semi-structured design is that the researchers could ensure the questions of interest were answered, but without restriction, allowing the participants the freedom to discuss whatever they wish, creating a more natural conversation, which can facilitate greater insight and understanding of participant perspectives [46].

Focus groups were audio-recorded using Dictaphones and recordings were transcribed verbatim, with all participant responses being pseudonymised at this stage [47]. These verbatim transcripts were imported into NVivo, where they were analysed primarily through inductive thematic analysis [48, 49]. Transcripts were coded by SW (Research Fellow), before discussing them with FS, SD (Study PI and Researcher, respectively) and then members of the wider team in a process of iterative theme development. This entailed multiple iterations of code and theme generation, in line with the six stages of analysis outlined by Braun & Clarke [50] – Familiarisation with the data; Coding; Theme Generation; Theme Review; Theme Definition; and Reporting. By SW leading analysis, it removed any personal biases that are likely to occur if this stage was conducted by the researcher who also led the focus groups [51, 52]. Where relevant there was additional interpretation of the latent implications within the explicit semantics [53, 54], with any subjective interpretations discussed, in order to get the opinion of multiple researchers. In addition to the inductive analysis planned, it became apparent when conducting the coding and theme generation stages that there was a clear resemblance and consistency between participant responses and the domains of the COM-B Model of Behaviour [55] and therefore, further ‘top-down’ deductive analysis was conducted [48, 49]. This meant that the analysis took a two-pronged approach, with both a data-driven initial stage of analysis, accompanied by a latter theory-driven derived coding [56].

Briefly, the COM-B Model [55] suggests that for a behaviour to occur, an individual must have adequate confidence in their Capability to perform the behaviour (physically and psychologically; i.e. do they have the knowledge and skills); their environment must allow them the physical and social Opportunity to do so (e.g. time, access, support, norms); and crucially, they must also have Motivation for it (can be automatic or reflective; e.g. emotions, habits, beliefs, conscious intentions). The model is now widely used across health psychology and behavioural medicine, aiding researchers to attempt to understand behaviour, and has been previously used on multiple occasions with regards to physical activity [57–59].

Ontologically, the analysis was approached with a critical realist outlook, partnered with an interpretivist epistemology, allowing us to accept that everyone will have experienced their reality differently, and thus their interpretations will have been impacted by their own individual experiences [60]. Overall, our aim was not to describe a social reality, but to explore the acceptability of the programme and connection experiences of our participants.

## Results

Three overarching themes were identified within the data, each with a number of subthemes categorised beneath them. The three themes were: Barriers & Facilitators to Engagement; Connection Pathway & Sign-Up Experience; and Golf for Health Programme Experience.

### Theme 1: Barriers & facilitators to engagement

This theme represents a frequently discussed element of the focus groups, regarding the barriers that participants faced, and how these were sometimes overcome or facilitated, predominantly in terms of the present Golf for Health programme, though some comments made could also be applied to general physical activity opportunities.

**Fig. 1:**
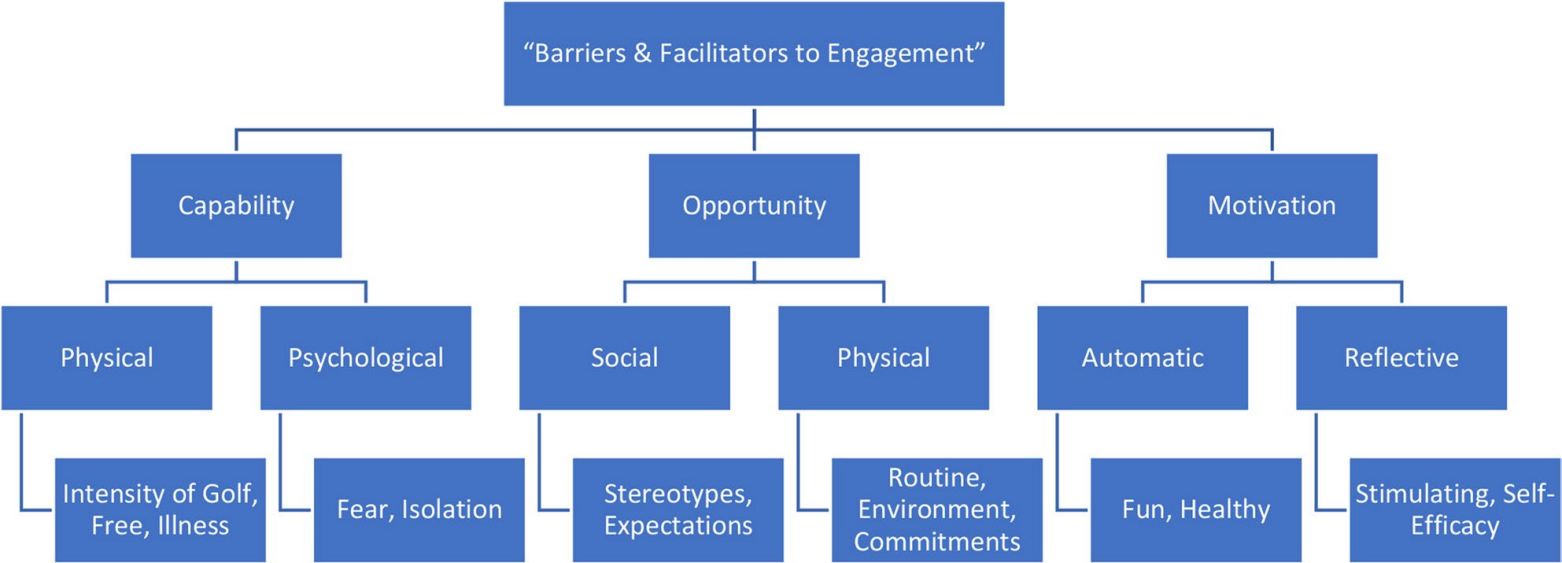
Thematic Map of Theme 1: Barriers & Facilitators to Engagement

Given the extensive range of responses, reflecting the individual nature of everyone’s experiences and their reason for attending, the subthemes within this overarching theme were created such that they were theoretically bound, in this case representing each domain and sub-domain of the COM-B Model of Behaviour [55], as demonstrated in Fig. 1. Whilst the theme heading was initially decided through iterative bottom-up thinking, it was decided that categorising the subthemes with a deductive, top-down approach enabled a more holistic and orderly view of the responses.

### Capability

Beginning with Capability (both physical and psychological aspects), as may have been somewhat expected amongst a sample connected with the opportunity for help with health issues and low activity levels, this was discussed as both a facilitator and a barrier to the programme.

From a physical perspective, some participants alluded to the low perceived intensity of golf as a physical activity, referring to it as “gentle exercise” (SP2), which was interpreted such that this enabled a sense of capability which they may not have had with other activities. In terms of how this impacts the likelihood of them subsequently performing the physical activity behaviour, the COM-B model suggests that by viewing golf as something that the individual *can* do, their confidence is enhanced, which is likely to increase the odds of engagement, both directly and also via increased motivation [61].

“*I can’t get up the whole length of the green now*,* I’ve not got enough energy*,* but I think I’d be able to swing a golf club” – WP3*

Psychological aspects were discussed even more frequently, with participants talking about experiencing issues with fear and anxiety about attending the sessions, as well as social isolation, which proved to be a barrier to participation. Again, this could have potentially been anticipated, given that the inclusion criteria were open to those with mental health concerns, and the programmes ran in the aftermath of the COVID-19 pandemic. Reflecting upon the impact of lockdown and anxiety, a time which impacted the mental health of many, following a period of enforced social distancing, isolation, and uncertainty [62, 63], one participant’s response was of particular note, recounting their feelings on the morning of the first session, further outlining the many thought processes that almost stopped them attending the programme:

*“I’ve been working from home since the pandemic and I’ve really not left the house very much other than you know*,* maybe to go and pick up prescriptions*,* so I’ve kind of hermitized myself a little bit …” – SP4*

The sense of fear and the unknown was clearly felt by the participant, who further went on afterwards to reiterate that they nearly “*talked themselves out of it”*, going on to highlight the anxiety of walking into a location and social situation of complete unknowns.

*“ There’s nothing more daunting than walking in somewhere-” – SP2*

*“-It’s that anxiety*,* it’s horrible” – SP4*

In the present case, it is fortunate that the individual mustered up the courage to attend irrespective of their concerns, but it is unknown how many people did not sign-up because of such fears. There was a process in place that if an individual reached out to the club ahead of their participation, the Professional who would be running the sessions would call to introduce themselves and answer any questions, to try and create a sense of familiarity and comfort. Though, it may have been beneficial that if a CLW was involved in the connection phase, this step had been done as standard, to facilitate the introduction and aid with that sense of comfort, to increase the individual’s sense of capability.

### Opportunity

The second sub-theme, Opportunity, was a further common topic of discussion. This represents comments made both in the remit of the social opportunity and also the physical, again including both barriers and facilitators to participation in the programme. The conversations that were classified as regarding physical opportunities were particularly centred around the notion that the programme provided the chance for structure and routine, which was an important facilitator for participants. Interestingly participants made the comparison to a work meeting, something that they had committed to and would attend without having to create the opportunity themselves. For some, this structure was required in order for them to take their physical activity seriously. However, for others this went further, and they did not see it just as exercise, but this gave them the opportunity to realise they can make time for their health and wellbeing, and set this as one of their priorities.

*“This is for me and I’m going to take that so*,* I’ve kind of got that confidence back*,* to say no you know*,* this is my time … it’s only an hour in the grand scale of things*,* in your day” –* SP5

Of course, this was not always possible and unsurprisingly, scheduling also proved to be a barrier, with people living busy lives, and as such having a variety of commitments each week. This was reflected by many in the focus groups, often being the reason that they felt they were unable to be active. This is not uncommon to see, and is frequently reported across the literature, with a perceived lack of time for physical activity one of the most prominent reasons for habitual inactivity, regardless of whether it is factual in reality [64–67].

“*I’m so busy*,* I have a fulltime job and I also run my own business as well so*,* that’s like pretty full on and I never stop . Maybe if there was an option of a bit more flexibility. I couldn’t make two of the weeks due to work commitments … My work are very good at giving you last minute meetings*,* first thing in the morning*,* can you attend this call and before you know it*,* they’ve taken your whole day*” – SP5

Other physical opportunity elements also showed themselves as barriers, particularly involving the environmental aspects. The weather proved an issue for the Autumn/Winter group especially, with participants reflecting on an occasion where the rain was too heavy for any activity to go ahead, with thunder and lightning in the area preventing any golf, noting that instead they sat in the clubhouse as a group and had a short social session with complementary hot drinks. It is worth noting that a social aspect was a key element for all groups, irrespective of weather – and so this was not unusual, but did prove to be a barrier to the activity aspect in this particular session. Conversely, another location also had similar issues with ‘*monsoon*’-like rain, but the group opted to utilise the shelter of the driving range and play anyway. This was not such an issue for the Summer iteration of the pilot however, who actually made reference to the fact that the time of year may have influenced their adherence and new-found enjoyment of golf, proving instead to be a facilitator - though they did also point out that the poor weather would have likely impacted their desire to participate.

*“If we didn’t like it*,* we wouldn’t have come back. It’s strange*,* I mean*,* if I was to take up golf*,* I wouldn’t want to play this time of year [Winter] for example. I’m a fair-weather sportsman*,* but that’s all. I think we’ve been very fortunate. The weather has been very kind to us.”* – SP3

Of course, not all the opportunity elements were physical in nature, with some of these barriers being socially aligned. For instance, whilst discussing how the programme gave them the physical opportunity to be active and learn golf, some of the participants were also then keen to justify themselves and why they were taking time away from work to attend the sessions. It became apparent that the viewpoints of others were important to the participants, and they did not want it to seem like they were not living up to their perceived social obligations and expectations by not being at work, despite the fact that it was essentially a prescribed weekly health appointment.

*“I never told people I’m away golfing because you know*,* they might turn round and get a bit arsey*,* they think I’m having fun … My boss knew*,* I told him and I said if you need any of the emails you know*,* to prove where I am*,* you know that kind of thing but I didn’t pass it on to anybody else because well*,* it’s none of their business” – SP5*

There was also pre-attendance concern about the environment they would be entering, with some of the participants wary of the stereotypes of golf as a sport, both in terms of being male-dominated and also expensive monetarily. An exchange between participants during Focus Group 1 discussing this showed clearly that these stereotypes very nearly proved to be a barrier to their participation, but one that actually became a facilitator upon the realisation of how welcoming and friendly an environment a golf club can be.

*“I almost never came*,* that morning everything was kicking off and you know*,* the whole I just don’t have the time and then it will be full of men*,* I thought I was going to walk into a room of men and I thought*,* do I really want to do this and then I was like no because I talk myself out of things all the time*,* you know*,* all the negatives start coming in you know*,* the fear and I thought no*,* just go for it” – SP4*

*“Actually you’ve also said something there that we’ve spoken about this morning. It was a very welcoming situation to single females. Now there’s definitely*,* I know if I think of other people who’ve spoken about other golf clubs*,* who find them less inviting*,* there’s a lot of males who are there to play the game*,* get on with it and not to have you get in the way and it was one of the reasons*,* I live very close to a golf club but I wouldn’t think of going there*,* for lots of reasons whereas [Club 4] definitely had a very*,* very different atmosphere and [NAME] said to me this morning*,* you would go on your own and not feel in any way intimidated” – SP3*

This proves to be something that requires consideration, especially when using golf as the exemplar in this research. Preconceived notions and stereotypes can clearly be enough to instil doubt and fear into potential participants - especially given that as mentioned, some of these participants had described experiencing fear and anxiety related to participation, and those with mental health concerns were not excluded. These doubts and fears could possibly impact adherence and motivation, and may have been detrimental to the connection phase, considering that at this stage individuals would not have known how welcoming the sessions were to be. Providing even the greatest opportunities and pathways can only be beneficial if the participant actually attends, and so ensuring that they get to the point of attendance is a large part of the struggle.

### Motivation

Finally, the third sub-theme of the ‘Barriers & Facilitators to Engagement’ theme regards the motivational aspects of participation, be that the reasons why people decided to start, or why they kept attending throughout. In line with the COM-B model, this reflected both automatic and reflective motivations. These automatic motivations are those sub-conscious thoughts and feelings, such as the immediate enjoyment they were having, and the innate desire to stay healthy. A number of the participants said how much they had enjoyed it, and the fun they had, which naturally was likely to have influenced their participation levels.

*“I’ve got to say I enjoyed what we did … probably I think for me*,* it hit the mark in terms of a gentle start*,* now I definitely still would like to continue” –* SP3

Regarding health, although it was often alluded to, most participants did not reference it directly without prompting. Instead, they were sharing the related outcomes that they hoped or anticipated they would achieve, acknowledging that the physical activity that this opportunity provided would benefit them and their health. Interestingly, there was more prevalent discussion around participants’ mental health rather than physical.

*“I need to try and get my stamina and fitness back”* – SP2

“*It is fun*,* but that wasn’t the intent it was to get me out you know*,* to get over my stress*,* anxiety*,* everything and obviously for me*,* it was something for me”* – SP5

There were also several instances of reflective motivation, resembling those conscious evaluations of their personal experiences and self-identity. Within the focus groups, this presented itself in various ways, including through the discussion of golf as a more mentally stimulating activity than walking alone, and how this made it a more appealing option for participants. Similarly, the idea of learning a new skill was seen as a positive. The notion of learning a new skill and the sense of improvement and mastery that can come with it is greatly beneficial for an individual’s self-efficacy [68, 69], which may have contributed to why some participants mentioned that their participation has led to feelings of increased confidence, including related to their confidence in a group setting. Furthermore, others alluded to how the participation has motivated them to do more activity and that they are already anticipating health improvements as a result.

*“I think it was just good coming and knowing we were learning-” –* WP2

“*I have to admit walking doesn’t appeal to me anymore*,* but walking around the golf course is the exercise you need but at least you’re doing something”* – WP5

Aside from these, it appears that the involvement of the NHS as a connection pathway and having their backing was also beneficial for motivating participants, with some reporting that they were under the impression that the NHS were the prominent party running and funding the programme. Responding to questions around the advertising of the project, one individual expressed their viewpoint that people may be more likely to participate if they were told to attend by a doctor, whilst another emphasised that the NHS involvement was deemed a strength.

*“I think that it’s quite a good way of pulling people in*,* the NHS thing and the health*,* that definitely gets people”* - SP3

*“If your doctor had said this is something you might be interested in*,* it would do you good*,* you probably would have had more interest in it then”* - WP2

### Theme 2: Connection pathways and sign-up process experience

The second theme was regarding that of the participant’s experience of the connection pathways and sign-up process. This theme covers all discussion surrounding what happened from the moment they were first informed about the programme, up to participation in the first session, with two distinct sub-themes.

When discussing the connection process, it naturally makes sense to explore the participant’s experience of the sign-up phase. No participant within either focus group had been connected directly by their GP, therefore this potential pathway could not be discussed. However, there were participants who had been connected by CLWs and Community Groups, which are often initially signposted to by GPs. Speculatively, this indirect connection may be a by-product of the current strain on GPs in the NHS, such that participants were unable to be seen, or the GPs were not able to take the time to connect in the short windows with which they saw each patient.

“*We used to have a men’s club and most of the men came and were referred from GPs just to interact more*” – WP4

“*People can’t even get appointments for GPs”* – SP4

In the present case, the most active engagement in connection with the programme taken by any facilitator was that of a CLW who directly sent an email about ’Golf for Health’ to their ‘patients’. Referring to the work of Cunningham et al., [15] to label this pathway, this was a case of a ‘Prescription followed by Active Signposting’, whereby the individual had been connected to an intermediary professional (CLW), who has then actively signposted the community-based physical activity opportunity to the individual. Similarly, many of the focus group participants were connected to the golf programme through a local health-based community group that they had initially been referred to via Primary Care, where the group leader was keen to suggest the programme to those they felt it would interest and helped them complete the application forms if necessary. This therefore translates such that both stages reflect connection/referral, with the Primary Care Professional referring the individual to the community group, who then actively connected them with the ’Golf for Health’ sessions. Other participants were connected via passive signposting, through the dissemination of the invitation poster displayed physically in GP Practices, or online via Practice Social Media.

*“I saw the poster at the doctors surgery*,* just in the waiting room”* – SP5

“*It was on Facebook*,* one of my friends had shared a post from [LOCATION] Practice and I was just scrolling and I just happened to see it and I was just in one of those sort of seize the day mode”* – SP4

Interestingly, given the quote above, a participant actually raised doubts about the effectiveness of a poster. Certainly from their own personal stance, it was suggested that without external intervention and recommendation, the poster would have been futile. This supports prior suggestions that direct professional advice may prompt patients to take health recommendations more seriously, especially by highlighting the potential risks of not following them [70].

“*If I’d seen it on a poster*,* I perhaps might not have thought the same about it*,* probably because somebody maybe is telling you about it and that sometimes comes across more than somebody when you’re reading it on a poster*” – WP2

However, there was further issue with the sign-up process that stemmed beyond the connection phase itself. Once participants had expressed their interest in the programme, it was not then an immediate process to starting the lessons, as the limited resources available during the pilot meant that all eight spots available needed to be taken before the programme would run in any given location. This led to confusion and impatience for two reasons. Firstly, those participants that were amongst the early cohort to sign-up had a potentially long wait for the other spots to be taken. Then even when these spots were taken, there was then a convoluted process that still needed to occur. Whichever the connection route taken to the programme, the individual would initially be taken to the ‘Golf for Health’ website to sign-up, and select their location of choice. Once the research team had confirmed their place on the programme, the club would contact the participants to ask what day/time suited them; collating all of the information, ensuring that there was an appropriate time and resources available; and then contact all of the participants again to check that they could do the agreed upon day/time.

This process was also deemed to be restrictive by the participants, as it meant that they had to decide the location they wished to attend right from the start, before knowing the time and day that the club would run their sessions, where another club may have been running their sessions at a more suitable time. Given that all five locations on offer were in Fife and within 40 miles of each other, it is possible that participants could have readily accessed multiple locations should they have needed to. During one focus group, participants actually suggested that if this is how it would work going forward, it should potentially allow the potential participants to give a priority ranking of locations.

Forming our second sub-theme, participants were also keen to raise their confusion around the eligibility criteria for the programme. It was stated on the advertisement poster that the programme was for “*NHS Fife patients who haven’t played before or haven’t played for quite some time*”. The *actual* eligibility criteria set for the programme was those who do not meet the current UK guidelines of 150 min of moderate intensity exercise per week AND who are new to golf, or who have not participated in a significant length of time (> 2 years) – however this was not necessarily clear from the advertisements. It seems as though naming the scheme ‘Golf for Health’, alongside the inclusion of ‘NHS Fife patients’ within the promotional material gave the impression that participants required a health condition.

“*I don’t know*,* when I read it*,* I thought you had to have some sort of illness or disability or you hadn’t done exercise*” – SP2

“*It says ‘speak to someone in your GP surgery*,* and sign up here today’. So it sounds like you could only sign up if you had spoken to somebody in your surgery”* – WP2

### Theme 3: Programme experience

This theme encapsulates the participants’ reported outcomes of their time in the scheme; the structure and programme schedule; and the purpose of the programme as a whole. As will be expanded upon, there were clear reports of both positive and negative aspects to the experiences of the individuals.

In terms of the outcomes of the programme experience, these took a few avenues, particularly reflecting the physical, mental and social health and wellbeing, as well as behaviour habits and outlooks of the participants. For instance, in terms of these health and wellbeing outcomes, a large proportion of reports were around the mental and the social aspect of this. Multiple participants referenced the increased confidence they felt they had gained from their participation. The importance of this is that not only will motivation likely be increased to perform future physical activity behaviours as a result of the heightened confidence, as highlighted in the quotes below, but also many of these participants were reporting to have mental health issues prior to engagement, and therefore an improved sense of social confidence may prove to be a big step for them in day-to-day life.

“*I guess some people were not likely to go out and play a hole on the golf course but I think we feel confident enough now that we could”* – WP5

A potential by-product of the enhanced confidence gained from their participation was the comfort they reported amongst their groups. However, it is unclear which was causal, as it was also reported by others that some of the comfort came from the fact it was a group setting rather than one-on-one lessons.

“*I would never have come along and just gone onto the golf course with just me or even somebody else*. *In a group it has given you confidence I think” – WP5*

This display of social connectedness and willingness to participate in group activities may have seemed an unlikely outcome at the start of the programme for some. Yet, such was the importance of this social aspect, and the subsequent comfort they had amongst each other, following the programme’s cessation, participants from one of the locations created a WhatsApp group chat amongst themselves so that they could continue to meet to play golf and remain friends. This was potentially the result of the diligent efforts of those running the sessions to establish a programme that was not solely all about golf lessons, but also ensuring that there was a welcoming environment and adequate opportunity to facilitate socialising.

“*What was really good was that last week*,* getting to see everybody and now we’ve got a wee WhatsApp group going and we can meet up for golf*,* I’m already doing that*” – SP4

This, in itself, was a significant and successful outcome of the pilot study. The fundamental aim of any programme of this type is that the participants become motivated to be physically active and carry on exercising even when the respective sessions are no longer part of this schedule. Therefore, it is a successful outcome that, when reflecting on their time in the scheme, participants were keen to stay active going forward, in terms of both golf and general activity - attributing this to the time they spent outdoors, and again mentioning the social aspect of the programme.

*“It’s encouraged me I think to be more active and to go out and get more fresh air because I just loved being outside*,* even though the weather wasn’t that great*,* it was just being outside. We met lovely people*,* it’s increased my enthusiasm for golf and I do know how to hit a ball now*,* maybe not properly” – SP2*

As stated, these positive outcomes were not all based on mental and social aspects, with some discussion also present surrounding improved physical health outcomes, and the concurrent behavioural changes. There were reports of motivational and volitional changes around peoples’ willingness to engage in physical activity. For instance, the following interaction in one focus group displayed the outlook of some participants, and how they felt they would use their experience. As might have been anticipated, some clearly caught ‘the golf bug’ and were very keen to continue, whilst others were placing more focus on their general health and fitness, and one participant was still seemingly a little apprehensive – but a combination is to be expected and is acceptable, as is noted with the present ontological and epistemological outlook.

*“Are you planning on doing it in the future*,* continuing?” - INT*

*“Maybe*,* yeah” – WP2*

*“Yes*,* I think so” – WP5*

*“Of course*,* if I get asked” – WP4*

*“I’ve got my golf clubs in the house*,* in the garage and I’ve taken some in and I was going to go and practice in my green because it’s got a big green*,* but it’s a bit too wet” – WP1*

Not every outcome was wholly positive, and despite golf being a low-impact sport, the golf swing does use a large amount of one’s muscle groups, with each stage of the swing having different muscular requirements from head to toe [71]. As such, it’s maybe unsurprising that participants did comment that in the early stages of the programme they were finding that they were aching afterwards, jovially remarking that after the first week participants were laughing amongst each other and stating,

*“We’ve come for our mental health but we’re going to go away with physical injuries”* – SP5

However, this quote also leans into our second subtheme within Programme Experience. The participant began to discuss the purpose of the programme as a whole, and their reason for joining, which proved to be something that was contended amongst participants, and a point of confusion for some. Despite their connection and willingness to sign-up and attend, it seemed that participants were unaware what they were actually meant to achieve from participation. This was not just in terms of not knowing what each session was going to be, but also why the programme was running as a whole.

*“It’s about expectations so … I think it was more about making us feel comfortable. [NAME] thought*,* she wondered if there was like a mental health aspect and they thought we were maybe there for mental health reasons*,* she wasn’t sure”* – SP2

*“I didn’t know what we were getting*,* I just turned up and hadn’t a clue as to how much golf we were playing or what we were going to achieve*” – SP5

This leads on to the final sub-theme, the Structure of the actual sessions themselves. It is possible that part of the reason that participants were confused about what service they were going to receive is that what would be expected or included was not standardised amongst the golf club staff running the sessions prior to the start of the programme. This led to extensive differentiation and low fidelity between sites, which became particularly apparent during the focus groups. In the locations represented in the focus groups, the first club was heavily golf-focused, with very-little social aspect reported; one club had lots of social time, but with far less golf than participants anticipated; however the final golf club discussed seemingly struck an appreciated balance of both golf tuition and socialising. This disparity was most explicitly captured in the following excerpt, however it is worth noting that those in the other focus group did report a more organised and gradual progression to their sessions:

*“I thought it was going to be more*,* golf*,* and it sounds like what you’ve had at [CLUB 1] is maybe what I thought it was going to be like you know*,* proper intensive tuition*,* golf tuition but I did look back at all the emails … and it actually says*,* it won’t be all about golf. Having fun*,* staying active and meeting new people is our goal”* – SP2

*“Ours was very much golf orientated … So*,* we went along*,* met the pro and then went straight to the pitch and putt course and the first week it was like*,* learning to do the swing for an hour and a half and how to hold the club*,* we just spent the whole hour and a half just doing that. We hit about*,* we must have hit about two hundred balls” – SP5*

There were other elements to the programme’s structure that were discussed too. The first relates in some capacity to the idea of social confidence that was previously discussed - group size. Participants across the board were pleased with the limited group sizes, feeling that this was not overwhelming for them in terms of anxiety in unknown social situations, but also when it came to the sessions, they felt that they were getting adequate attention, citing *“You wouldn’t get much input or training if it was too big a group”* – SP5.

In terms of strategies and offerings for when the programme finished, it was planned that participants would be offered some form of special continuation scheme by their respective clubs – though these were not set in stone and were at the discretion of the golf club. Despite the general accessibility of golf in Scotland, it was important that participants were not met with unexpected barriers at the end of the scheme. Overall, irrespective of any location-based differences, it was clear that a continuation scheme was desired by the participants, but the timing of the focus groups being very soon after completion meant that these had not yet been officially offered.

“*If you’re doing it for the eight weeks and you really want to continue it*,* then there needs to be some way to enable you to do that” –* SP4

## Discussion

The present study utilised focus groups of a sample of participants in a local ‘Golf for Health’ programme to explore its acceptability, showing that connection to this opportunity could prove to be beneficial and desired, but that the current model may need development. Firstly, and most importantly, the participants reported to enjoy their participation, felt that they had benefited from it and some explicitly highlighted their desire to continue to play golf. Additionally, the focus groups enabled the researchers to gain vital insight into the experience of the participants. It was clear that whilst mostly positive, there were issues that participants raised regarding the experience, as reflected by the ‘Barriers’ labelled throughout, especially in Theme 1, the sign-up process in Theme 2, and the contention around the anticipated programme purpose in the final theme.

As highlighted in depth within Theme 1, despite best efforts, the Golf for Health programme was not immune to barriers to engagement, but did also provide a number of facilitating factors – as the design intended. It is clear that Capability, Opportunity and Motivation were all attributable factors that were present in the experiences of the participants, however their own individual experiences determined whether these were in the most-part positive or negative.

Moving forward, exercise programmes such as this should ensure that participant feedback is taken on board and developed, such that those elements that were reported to be facilitators are emphasised and maintained, whilst work must be done to ensure that those barriers that can be addressed. It is only through listening to participant feedback that programmes can develop appropriately for greater implementation outcomes, to enable progression [72].

This focus group study can now provide vital PPI insight for the development of the programme, building on the pre-programme perceptions and anticipations [39]. For instance, in terms of Capability we can ensure that those running the sessions do not make it overly intensive, enabling participants to develop the sense of physical capability, whilst also developing their skills to a level whereby they are confident that they can perform the behaviour. Furthermore, the premise of the entire programme is enabling participants the physical opportunity to participate in the activity, though perhaps more emphasis should be placed on highlighting that it is for health purposes and so more effort should be made to see it this way. Finally, regarding the Motivation element of the programme, the direct facilitative avenues present in the current programme are such that not only is participation fun, but also reminding participants that it is beneficial for their health, as well as ensuring that they have a real sense of mastery and achievement, which will likely increase self-efficacy and therefore motivation to maintain the behaviour.

The connection pathway remains a key aspect and interest of the overall project, and is something that if this programme, or any similar such local physical activity opportunities, are to be successful and ecologically valid, must be effective. Without a natural, smooth and efficient process from community healthcare settings to these opportunities, the likelihood of patients utilising them is minimal – hence the need for the research to occur in the first place. In this case relevant stakeholder engagement was present, however in need of fine-tuning, with participants explicitly highlighting their feelings around the current climate with GP appointments being difficult to attain [73]. Therefore, not only does the Golf for Health programme need to be efficient and clear for all forms of Healthcare worker (given the potential connection pathways via intermediaries such as CLWs), but also ensure that the process itself is as smooth as possible, ensuring that wait times are not excessive, and that the programme is as accessible as it should be. As became clear in this research, and in the stakeholder engagement sessions in conceptualisation [39], we cannot expect GPs to be the sole connection pathway, and we must take advantage of the other vast connection options available, but we should still utilise their help to support and facilitate the process. In addition to a smooth process for the initial connection step, it became apparent that participants felt that in some instances the time taken between connection and commencing was too long. This delay was the result of recruitment issues, and the requirement to reach certain registration numbers before the programme would start. Therefore this may also be something that was specific to the circumstances, when it is considered that this was a new programme, and so it is possible that knowledge of the opportunity was not widespread, or that there may have been hesitancy around a new ‘unknown’ opportunity.

Aside from successfully gaining the insights of participants, amongst the strengths of this research, the ability to naturally underpin the responses to theory proves to be a positive. Doing so enabled claims made to be grounded within behaviour change theory – which subsequently enables the comparison and amalgamation of this information with that of others in the field. Additionally, we collected data at two points in time, reflecting both of the time-points that the programme was conducted (i.e. Spring and Autumn/Winter), providing a holistic representation of participants’ experiences.

From a further methodological viewpoint, the researcher who conducted the focus groups was not involved in the thematic analysis, and therefore there was no researcher-bias in either the way the focus groups were conducted nor the analysis. Furthermore, though not initially considered within the design phase of the study, the ability to retrospectively apply the Consolidated Criteria for Reporting Qualitative Research (COREQ) framework also proves a strength [52]. This checklist acts as an aid to improve transparency of qualitative reporting, particularly for interviews and focus groups. The successful application highlights the positive methodological considerations that were made, and bolsters credibility. Additionally, this represents another clear indication of the importance that the research team has placed on PPI engagement in the development of the programme, in tandem with Carstairs et al., [39] providing pre- and post-programme input.

Although, there were also limitations to the research. Our sample size reflected less than 20% of those who signed-up to the programme, and therefore there is potential response bias, such that the sample across our focus groups were not wholly representative of all participants. However it should be noted that 23 participants opted for survey feedback instead of focus group. Focus groups can also be susceptible to a group bias, especially in a setting such as ours where some participants have reported to have anxiety, therefore increasing the likelihood of them agreeing with the others in the group to form a norm rather than reflecting the entirety of their own opinion [74] – though this may alternatively have proved to be strength of our use of smaller group sizes, as it limits the chance for group-norm formulation, or pressure to conform. This may have been furthered by the presence of the coach in one focus group, as although they provided interesting insight, those participants who were taught by them may have felt pressure to be positive about their experience. Similarly, the presence of a long-term health condition may be of note here, as participants all reported having one, and this may have had a significant impact on their perceptions of barriers [5, 6].

Additionally, methodologically speaking, analysis was predominantly conducted by just one researcher, who created all of the initial codes and draft themes before corresponding with other members of the research team to undergo the reflexive aspects of the thematic analysis process. Moreover, applying the COM-B Model to the themes added an extra layer of complication, such that qualitative themes and sub-themes are often expected be derived to be entirely stand-alone without overlap, though this is inherently difficult when using a behaviour change model theory that encourages and emphasises the necessary interplay between each of its domains and sub-domains.

Whilst older age and presence of a long-term health condition were not prerequisites for participation, we had a significant proportion of our participants (in the programme, and the focus groups) who met both of these. With an average age of 64, and all participants in these focus groups having a long-term health condition, it is possible that these participants may have experienced the programme differently to a younger participant who was largely healthy, but otherwise inactive (and thus still met the inclusion criteria). For instance, in terms of physical fitness benefits, it may be anticipated that younger individuals will start at a higher baseline, and therefore older adults have the potential to experience a greater relative increase (i.e. in cardiovascular efficiency or muscle power; [75]). Previous research has also highlighted differences in exercise motivations between age groups [76], which has been suggested to influence adherence rates [77, 78]. Furthermore, exercise has been postulated as a potential aid in the management of chronic disease, reducing mortality rates, improving quality of life, and alleviating symptom severity [79].

### Future directions and recommendations

The findings from this evaluation suggest several key areas for refinement in the design and delivery of future golf-based social prescribing interventions.

1. Diversifying Referral Pathways
  Feedback from participants and stakeholders highlighted that reliance solely on GP practices and direct signposting is insufficient as the primary connection mechanism (Theme 2). Future programmes should therefore adopt a multi-channel referral strategy that integrates broader primary care networks, allied health professionals, and community organisations. Additionally, provision for self-referral facilitated through passive signposting and public awareness initiatives, may enhance accessibility and inclusivity, ensuring that individuals who might not regularly engage with primary care services are also reached.
2. Streamlining the Enrolment Process
  The current multi-step registration process was perceived as inefficient and potentially discouraging for participants. Although participants did not explicitly identify automation as a solution, future schemes could benefit from implementing an online enrolment system that allows golf clubs to predefine session times and days. This would enable prospective participants to select locations based on convenience, reducing administrative delays and improving overall efficiency. Simplifying the enrolment process may also improve initial engagement and reduce attrition during the connection phase.
3. Enhancing Participant Flexibility and Autonomy
  Participants expressed a clear desire for greater flexibility in session timing and location. Incorporating this flexibility at the point of enrolment through a digital system where participants can view available sessions and rank preferred venues, could strengthen adherence and long-term engagement [80].
4. Standardising Programme Structure Across Locations
  It became evident that there was a lack of fidelity across the programme locations, with participants sharing the disparity in session content (as highlighted in Theme 3). As a result of this, alongside representatives from the PGA of Scotland and the R&A, we have since created a specific structure for future iterations to follow, that utilises the Golf Foundation's coaching techniques, but still allows flexibility dependent on location facilities. The importance of fidelity across programme locations when evaluating effectiveness is vital [79]. This can also assist with allowing an extra level of flexibility.
5. Embedding a Structured Welcome and Peer-Support Component
  Fear and anxiety were recurrent themes influencing both engagement and retention (Theme 1). A formalised welcome or “buddy” scheme should therefore be embedded as a core component of future interventions. Pairing new participants with experienced peers or facilitators may help mitigate apprehension, foster social cohesion, and promote a sense of belonging, key elements in sustaining participation within community-based programmes.
6. Consolidating Learning Through a Transferable Implementation Toolkit
  Building upon the insights gained, a comprehensive “Community Golf Programme Toolkit” is currently being developed in partnership with The R&A. This resource will synthesise operational guidance, recommended practices, and identified pitfalls, offering a replicable model for practitioners and policymakers seeking to design or scale similar initiatives.

## Conclusion

Golf for Health has significant potential to offer an accessible and social introduction to golf, which could prove to provide long-term health and wellbeing benefits for patients. The participants of the pilot iterations reported to enjoy their experience, and that they had benefitted from their participation too, highlighting their notable acceptability of the programme. When reflecting upon the scheme using the COM-B Model, it was apparent that there were a number of facilitators to engagement present, that address many of the frequently reported barriers to exercise. Nonetheless, there is evidently still more work needed on developing and optimising the programme, particularly in terms of ensuring that we utilise connection pathways that are acceptable and feasible in practice, from the participant viewpoint.

## Supplementary Information

Supplementary Material 1.

## Data Availability

The pseudonymised transcripts that form the research data supporting this publication can be accessed at (10.17630/a1b8fe48-6ee7-4b2d-9d9f-7947bde60b63).
