# Supplementary material for: “Walking around the golf course is the exercise you need”: Exploring the acceptability of a golf on referral scheme amongst participants through post-programme focus groups
Source: BMC Prim Care. 2026 Jan 27;27:108. doi: 10.1186/s12875-026-03180-1 (PMC13032435; doi:10.1186/s12875-026-03180-1)
Supplement: Supplementary file 1 — Supplementary Material 1. [file 12875_2026_3180_MOESM1_ESM.docx]

## **Focus Group Topic Guide and Demographics**

Connection Stage to Golf for Health

How were you connected with/did you hear about/did you get involved in *Golf for Health*?

What worked/could have been better?

What other organisation would you like to hear about initiatives such as Golf for Health from?

The Registration Form

Did you complete the registration form on your own?

How did you find this?

What could have made it easier?

The Programme

Why did you attend golf for health?

What first appealed to you about the programme?

Did you get out of the programme what you expected?

Were there any issues or barriers to attending?

How did you overcome these?

What helped you make the decision to attend?

What benefits has there been to you in attending the programme? (health, social)

What did you like/dislike about the programme?

Did the timing/length/location suit you?

Any other comments or questions
